# Ultra-widefield confocal imaging of multiple evanescent white dot syndrome

**DOI:** 10.1016/j.ajoc.2020.100861

**Published:** 2020-08-06

**Authors:** Shin Kadomoto, Akihito Uji, Yuki Muraoka, Akitaka Tsujikawa

**Affiliations:** Department of Ophthalmology and Visual Sciences, Kyoto University Graduate School of Medicine, Kyoto, Japan

**Keywords:** Multiple evanescent white dot syndrome, Confocal color scanning laser ophthalmoscope, Ultra-widefield imaging

## 1 Case report

**Fig. 1:**
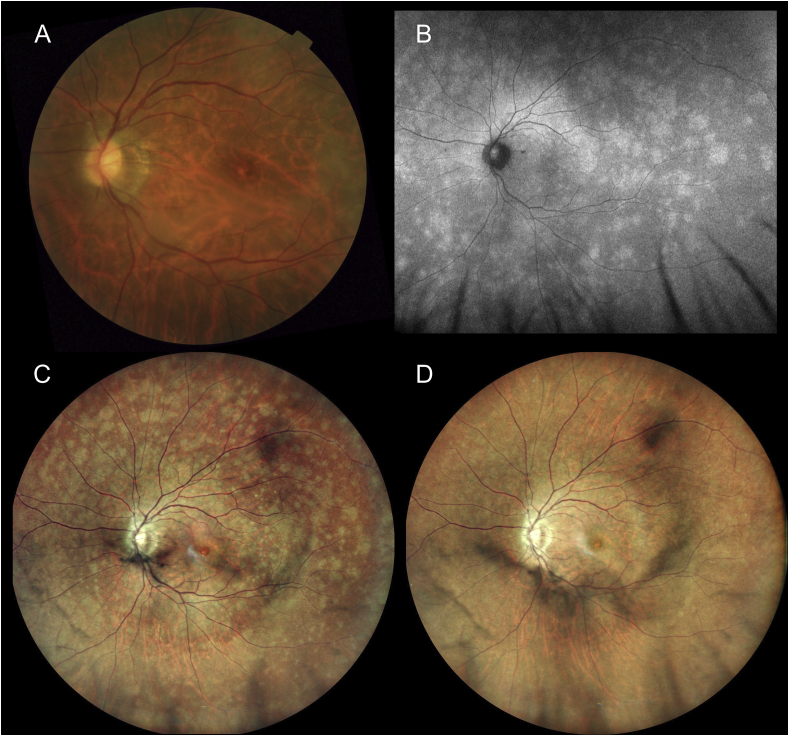
A: Color fundus photography (CFP) image. Retinal abnormalities are not clearly observed in this image. B: Ultra-widefield autofluorescence image (Optos 200Tx, Optos PLC, Dunfermline, United Kingdom) at initial visit revealing multiple hyperfluorescent dot lesions spreading to the mid-periphery of the retina. C: Ultra-widefield confocal scanning laser ophthalmoscope (SLO) image (Mirante, Nidek, Gamagori, Japan) at initial visit. The white dot lesions are clearly visualized compared with CFP(A). D: Ultra-widefield SLO image two weeks after the initial visit. The white dot lesions have disappeared. . (For interpretation of the references to color in this figure legend, the reader is referred to the Web version of this article.)

A 49-year-old woman with myopia presented with blurred vision in her left eye. The vision was 20/25 in her left eye. She had a few vitreous cells at the initial visit. Color fundus photography (CFP) (Fig. 1 A) showed a few ill-defined white dot lesions. However, ultra-widefield autofluorescence image (Fig. 1 B) and confocal color scanning laser ophthalmoscope (SLO) image (Fig. 1 C) revealed multiple white dot lesions in the posterior pole and at the mid-periphery of the retina. Compared to CFP, ultra-widefield confocal color SLO delineated white dot lesions with high contrast. The patient was diagnosed with multiple evanescent white dot syndrome (MEWDS). After observation for two weeks, her vision recovered to 20/20 and the white dot lesions completely resolved (Fig. 1 D).

## 2 Discussion

Multiple evanescent white dot syndrome (MEWDS) is a unilateral, acute, multifocal retinopathy affecting young to middle-aged adults.^1^ Numerous small white dot lesions are usually observed ophthalmoscopically in the paramacular area spreading beyond the vascular arcade. The visualization of white dot lesions with an ophthalmoscopic device is important for the diagnosis of MEWDS and for observing the evolution of lesions over time. Color fundus photography and autofluorescence images are considered to be the best non-invasive modalities to assist the ophthalmologist in visualizing white dot lesions.^2^ However, it is sometimes difficult to find white dot lesions at the peripheral retina and in the paramacular area in terms of image contrast in CFP.

A confocal optical system reduces any scattered or reflected light outside the focal plane, providing high contrast and high resolution images compared to traditional fundus camera imaging.^3^ In this case, ultra-widefield confocal color SLO showed high contrast white dot lesions from the paramacular area to the mid-periphery of the retina corresponding to the autofluorescence image. The lesions were not clearly observed using CFP. Confocal color SLO images may be comparable to autofluorescence images with higher image contrast compared to conventional CFP.

## 3 Conclusions

Ultra-widefield confocal SLO imaging may improve diagnostic acumen and ongoing monitoring in MEWDS.

## Patient consent

Consent to publish this case report has been obtained from the patient in writing.

## Funding

No funding or grant support

## Authorship

All authors attest that they meet the current ICMJE criteria for Authorship Acknowledgements:None.

## Declaration of competing interest

The following authors have no financial disclosures: SK, AU, YM, AT.
